# Pattern of blood utilization and blood wastage at blood bank of a tertiary care hospital in Lahore

**DOI:** 10.12669/pjms.42.8.13958

**Published:** 2026-08

**Authors:** Asma Akhtar, Naureen Saeed, Ahsen Muzaffar, Zunairah Mughal

**Affiliations:** 1 Dr. Asma Akhtar, MBBS, FCPS Hematology, DHEP. Assistant Professor Hematology , Shalamar Medical & Dental College, Shalamar Institute of Health Sciences, Lahore, Pakistan; 2 Dr. Naureen Saeed, MBBS, FCPS Hematology. Associate Professor Hematology, Shalamar Medical & Dental College, Shalamar Institute of Health Sciences, Lahore, Pakistan; 3 Dr. Ahsen Muzaffar, MBBS Senior demonstrator Hematology, Shalamar Medical & Dental College, Shalamar Institute of Health Sciences, Lahore, Pakistan; 4 Dr. Zunairah Mughal, MBBS, FCPS Hematology Senior demonstrator Hematology, Shalamar Medical & Dental College, Shalamar Institute of Health Sciences, Lahore, Pakistan

**Keywords:** Blood Transfusion, Blood Banks, Blood Component Transfusion, Blood Preservation, Blood Utilization, Blood Wastage, Blood Donors

## Abstract

**Objective:**

To analyze pattern of blood component utilisation and determine the extent and causes of blood wastage in the blood bank of a tertiary care hospital in Lahore.

**Methodology:**

A retrospective observational study was conducted at the Blood Bank of Shalamar Hospital, Lahore, from January 1, 2022 and December 31, 2023.Board. Data on the number and type of blood components prepared, issued, and discarded were retrieved from blood bank record. Causes of blood wastage, including expiry, transfusion reactions, delayed return after issuance, broken bags or seals, and haemolysed or clotted units, were reviewed. Data were analyzed using SPSS version 24, and results were expressed as frequencies and percentages.

**Results:**

A total of 9,406 blood donations were collected. Of these, 8129 units were issued to clinical departments, while 1,277 units were discarded, resulting in wastage rate of 13.5%. Whole blood (41.8%) and red cell concentrates (36.8%) were most frequently utilized. The Intensive Care Unit (21.4%), Gynecology and Obstetrics (19.9%), and Medical units (16.7%) were the leading end users. Expired units accounted for 96% of blood wastage, with fresh frozen plasma forming the largest proportion (45%).

**Conclusion:**

Whole blood and red cell concentrates were the most commonly used components. Blood wastage exceeded international benchmarks, with expiry as the predominant cause. Regular audits, rational ordering, and adherence to transfusion guidelines are essential to optimize utilization and minimize losses.

## INTRODUCTION

Blood is a vital resource that plays an essential role in many lifesaving situations, including trauma, major surgery, chemotherapy, severe anemia, and obstetric complications. Access to safe and screened blood products is therefore of paramount importance.^1^ Ensuring an adequate and safe blood supply is particularly challenging in developing countries, where there is a shortage of voluntary blood donors, limited facilities for component preparation and storage, and inappropriate ordering and utilization practices.^2^

In addition to unnecessary requests and limited use of component therapy, wastage of blood products accounts for the loss of an estimated five million units each year. Several reasons for wastage have been highlighted in the literature, including expiry of blood bags due to non-utilization during shelf life (60%), broken seals (30%), returned bags after dispatch (13%), clotted or haemolysed units (4%), and reactivity for transfusion-transmitted infections (TTIs) (50%).^1^,^3^ In the United States, the annual wastage rate ranges from 1% to 5%,^3^ whereas developing countries report much higher rates of 16–25%.^4^-^6^

Pakistan requires approximately 1.2–1.5 million blood units annually, but continues to face a shortage of nearly 40%. Wastage is one of the leading contributors to this shortfall.^7^ Monitoring of blood utilization and wastage remains neglected at the national level due to suboptimal standards of blood bank practice and the absence of structured transfusion service audits.^8^ By contrast, developed countries have successfully reduced wastage rates to below 5% through systematic identification of underlying causes and targeted preventive measures.^9^

Limited studies are available on blood utilization and wastage patterns in tertiary care hospitals of Pakistan, and existing reports demonstrate considerable variation in wastage rates.^1,6.^ This highlights the need for regular monitoring and evaluation to guide corrective measures. Therefore, the present study was designed to review and analyze blood utilization patterns (whole blood and components) and wastage at the blood bank of a tertiary care hospital in Lahore, with the objective of identifying areas where targeted interventions can improve utilization and minimize losses.

## METHODOLOGY

A retrospective observational study was conducted at the Blood Bank, Department of Pathology, Shalamar Hospital, Lahore. Transfusion records from the Blood Bank January 1, 2022 and December 31, 2023 were reviewed to evaluate patterns of blood utilisation and wastage.

### Ethical Approval:

It was obtained from the Institutional Review Board of Shalamar Medical and Dental College, Lahore (IRB/AL/2024-030; dated April 17, 2024),

Data were included for all blood products collected, issued, and discarded during the study period. Donor screening was performed in accordance with World Health Organization (WHO) guidelines, including completion of a donor history questionnaire, physical examination, haemoglobin estimation, and testing for transfusion-transmissible infections (TTIs) such as hepatitis B virus, hepatitis C virus, human immunodeficiency virus, syphilis, and malaria using Chemiluminescent Immunoassay (CLIA). Donors with a history of high-risk behavior, jaundice, intravenous drug use, tattooing, recent surgery, or previous blood transfusion were excluded from donation. Records with incomplete, duplicate, or erroneous entries were excluded from the analysis

A structured proforma was used for data collection. The variables recorded included total donations, types of components prepared such as Red Cell Concentrates (RCC), Fresh Frozen Plasma (FFP), and platelets, number of units issued to various clinical departments, and details of wastage of blood units due to expiry, delayed return after 30 minutes, transfusion reactions, or physical damage such as broken seals or hemolyzed or clotted units. Transfusion reactions were defined as any adverse event documented during or following transfusion that resulted in non-utilization or disposal of the blood component. Blood units returned to the blood bank more than 30 minutes after issue were considered unsuitable for reissue in accordance with institutional transfusion practices and were categorized as wastage. Data cleaning was performed to verify completeness and accuracy before analysis. The overall blood wastage rate was calculated as the percentage of discarded blood units relative to the total number of blood donations collected during the study period. The final dataset was analyzed using SPSS version 24, and categorical variables were expressed as frequencies and percentages.

## RESULTS

Between January 2022 and December 2023, a total of 9,406 blood donations were received at the Blood Bank of Shalamar Hospital. Of these, 8,129 units were issued to various clinical departments, while 1,277 units were discarded; resulting in an overall blood wastage rate of 13.5%

Whole blood was the most frequently issued component (41.8%), followed by red cell concentrates (36.8%). The pattern of blood utilization across different hospital wards is summarized in Table-I.

**Table-I:** Distribution and Utilization of Blood Products across Hospital units.

No of blood units issued from blood bank	8129 units
No of Whole Blood units issued	3396(41.8%)
No of RCC issued	2990(36.8%)
No of FFP issued	1337(16.4%)
No of Platelets (Random donor +Single door) issued	406(5.0%)
*Ward-wise Utilization of RCC and Whole Blood (n = 6386)*	
ICU	1364(21.4%)
Gynecology & Obstetrics ward	1270 (19.9%)
Medical ward	1068(16.7%)
Surgical Ward	882 (13.8%)
CCU	667 (10.6%)
Pediatrics Ward	556(8.7%)
Orthopedic ward	403(6.3%)
Miscellaneous (Medicine & surgery allied)	166(2.6%)

**Fig.1:**
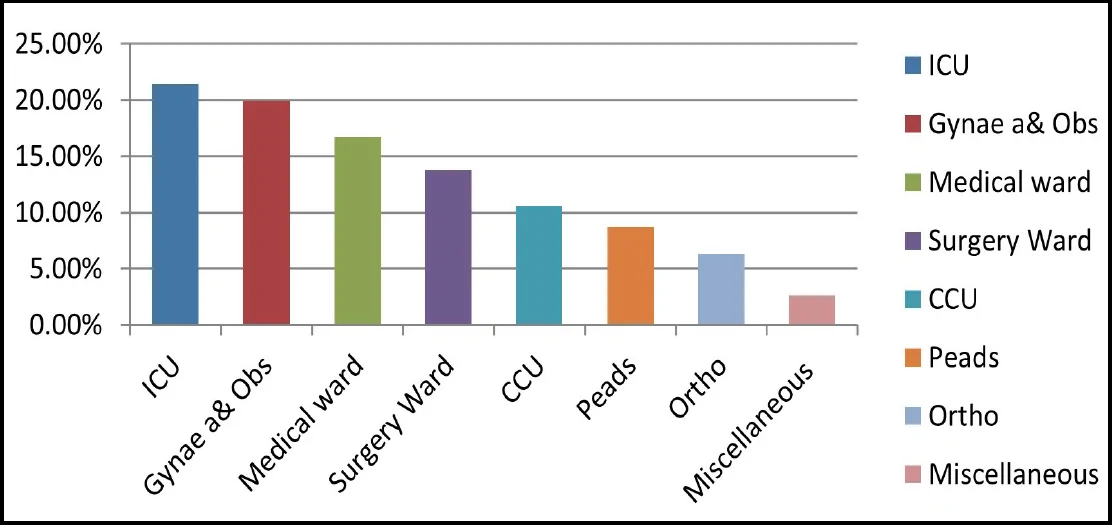
Ward-wise Pattern of Blood Product Utilization.

Among the 8,129 blood units issued, whole blood (WB) and red cell concentrates (RCC) accounted for the majority (n = 6,386; 78.6%). These were primarily utilized by the Intensive Care Unit (21.4%), followed by the Gynecology & Obstetrics ward (19.9%) and Medical units (16.7%). The detailed distribution of RCC and WB usage in these areas is shown in Fig.1 and Table-I.

**Fig.2:**
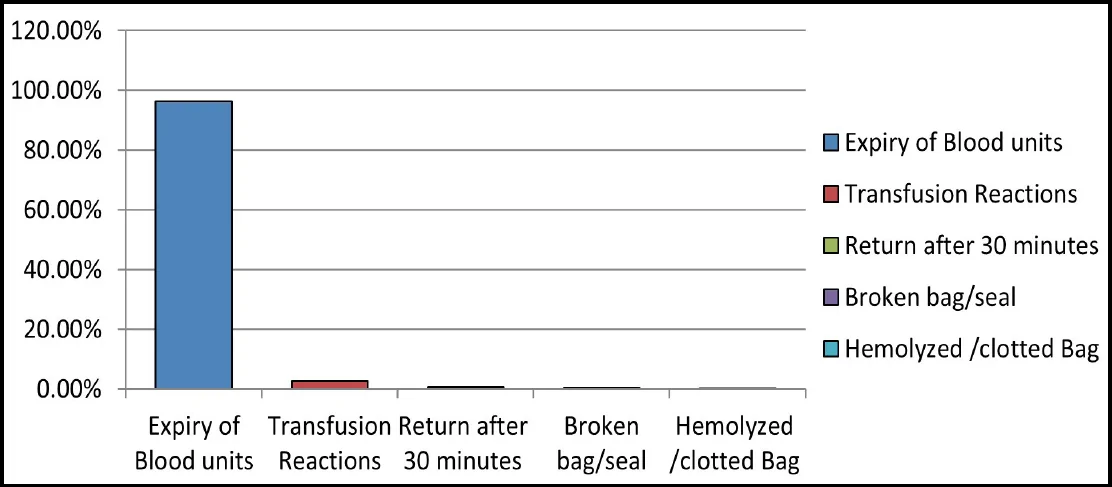
Reasons for Blood Product Wastage.

Out of 9,406 collected blood donations, 1,277 units were discarded, reflecting an overall wastage rate of 13.5%. Fresh frozen plasma (FFP) accounted for the largest proportion of discarded units (575; 45.0% of total wastage), followed by whole blood (354; 27.7%) and red cell concentrates (347; 26.7%). Platelet wastage was negligible, accounting for only one discarded unit (0.06%). As shown in Table-II and Fig.2, expiry of blood units was the predominant cause of wastage (96.28%), whereas transfusion reactions, delayed return of blood units, and physical damage contributed minimally.

**Table-II:** Overview of Blood Product Wastage and Component-wise Distribution of Discarded Units.

Total No of Blood Bags Wasted	1277 units (13.5%)
***Component-wise Distribution of Discarded Units***	
FFP	575 (45% of total wastage)
Whole Blood	354(27.7% of total wastage)
RCC	347 (26.7% of total wastage)
Single Donor Platelets	01 (0.06% of total wastage)
***Reasons for Blood Wastage***	
Expiry of Blood units	1230 (96.28%)
Transfusion Reactions	33(2.63%)
Delayed Return of blood units	07 (0.54%)
Broken seal of blood unit	05 (0.40%)
Hemolyzed /clotted Bag	02 (0.17%)

A comparison between 2022 and 2023 demonstrated improved blood utilization and reduced wastage during the study period. While blood donations and utilization increased in 2023, the overall wastage rate declined compared with 2022 (Table-III).

**Table III:** Comparison of Blood Utilization and Wastage between 2022 and 2023.

Parameter	2022	2023
Blood donations collected	4,481	4,925
Blood units issued	3,716	4,874
Blood units discarded	765	512
Blood wastage rate (%)	17.1	10.4

## DISCUSSION

The efficient use of blood and its components is fundamental to patient safety. The World Health Organization identifies rational use of blood as a cornerstone of safe transfusion practice, yet low- and middle-income countries, including Pakistan, continue to face challenges such as excessive ordering, inappropriate transfusion requests, and limited adoption of component therapy. According to international benchmarks, blood wastage should be kept below 5%, a target that many centers in developing regions struggle to achieve. In response, Pakistan’s National Blood Policy advocates for reducing reliance on whole blood and promoting evidence-based component therapy to ensure optimal utilisation of resources and minimize wastage^10^,^11^

In our study, a total of 8,129 blood units were issued during the study period. WB (41.8%) and RCC (36.8%) together accounted for 78.6% of transfusions. These were mainly utilized in the ICU (21.4%), Gynecology and Obstetrics ward (19.9%), and Medical ward (16.7%). Other low- and middle-income settings have reported utilisation patterns comparable to ours.

At Jimma Medical Center, Ethiopia, WB accounted for 96.6% of all transfusions, while RCC, platelets, and FFP represented 1.9%, 0.7%, and 0.5%, respectively; most transfusions were administered in the Medicine department (48.8%), followed by Surgery and the Gynecology and Obstetrics departments.^12^ In comparison, WB use in our study was lower (41.8%) yet remained high relative to international standards, with the ICU (21.4%) and Gynea & Obstetrics (19.9%) identified as the leading end users.

Studies from India and Saudi Arabia have reported greater reliance on blood component therapy, particularly red cell concentrates (RCC), which accounted for 42–84.6% of transfusions. Medical, Obstetrics and Gynecology, Surgery, Orthopedics, and Intensive Care Unit services were reported as the major end users of blood components in these settings.^13^-^15^

Within Pakistan, data from Hayatabad Medical Complex showed that the Paediatrics department utilized 21.2% of blood components, followed by Obstetrics (19.1%) and Medicine (16.7%). Whole blood (WB) was the most frequently transfused product, followed by red cell concentrates (RCC), showing utilisation patterns similar to those observed in our study.^16^

Taken together, these comparisons reveal that while most international centers prioritise component therapy, particularly RCC, hospitals in developing regions including ours continue to rely heavily on whole blood transfusion. The utilisation pattern in our study, driven mainly by ICU and Obstetric demand, underscores the need to strengthen component based transfusion practices and promote evidence based transfusion strategies through regular audits and clinical education.

In our study, out of 9 406 collected blood donations, 1,277 units were discarded, giving an overall wastage rate of 13.5%. FFP was the most frequently wasted component, accounting for 45% (n = 575), followed by WB 27.7% (n = 354) and RCC 26.7% (n = 347). Expiry of components was the predominant cause of wastage, contributing 96.3% (n = 1 230), while transfusion reactions (2.6%), delayed return of issued bags after 30 minutes (0.5%), broken seals (0.4%), and haemolysed or clotted units (0.2%) accounted for a small proportion. No unit was discarded due to TTIs, reflecting the blood bank’s strict pre-donation screening policy using the Chemiluminescence Immunoassay (CHLIA) technique, which has an approximately 99% sensitivity. A year-wise comparison revealed a reduction in blood wastage from 17.1% in 2022 to 10.4% in 2023. Although the specific factors responsible for this improvement were not evaluated, the finding may reflect improvements in inventory management, component inventory monitoring, stock rotation practices, demand forecasting, and increased emphasis on the rational use of blood and blood components. This encouraging trend highlights the potential value of regular audits and continuous quality improvement initiatives in optimizing blood bank performance and reducing blood wastage.

Compared with findings from Taiwan, India, and Libya, the blood wastage rate observed in our study was higher than that reported from Taiwan and India but lower than that reported from Libya.^17^-^19^ Fresh frozen plasma accounted for the largest proportion of discarded units in our setting, whereas other studies identified platelets or red cell concentrates as the most frequently discarded blood components. These differences may reflect variations in blood inventory management practices, transfusion policies, demand patterns, and the availability and utilization of blood components across institutions. Given that expiry of blood components was the leading cause of wastage in our study, measures aimed at improving inventory control, stock rotation, demand forecasting, and component utilization may help reduce blood wastage.

In the United States, Nguyen et al. observed plasma as the most frequently wasted component.^20^ This closely paralleled our experience, where FFP wastage predominated and expiry was the overwhelming cause. Together, these comparisons highlight the wide variability in global wastage patterns and emphasize the need for stronger inventory control, better demand forecasting, and patient blood management strategies in our setting.

Within Pakistan, reported wastage rates have shown considerable variation. At the National Institute of Blood Diseases (NIBD), Karachi, an overall wastage rate of 3.5% was reported, with FFP as the main discarded component, primarily due to component expiry and bag breakage.^1^ A similar rate (3.5%) was observed at Aga Khan University Hospital, Karachi, where FFP was again the most frequently discarded component, mainly due to expiry (59%) and bag breakage (29%).^3^ These findings are comparable to our results. At Liaquat National Hospital, Karachi, the reported wastage rate was higher at 10.9%; among discarded units, 5.6% were rejected due to TTI positivity, while platelet concentrates accounted for the largest share of wastage, primarily due to expiry.^6^ At the Blood Bank of Rehman Medical Institute, Peshawar, a wastage rate of 5.13% was documented; platelets showed the highest wastage (7.99%), followed by RCC (4.84%), cryoprecipitate (3.23%), and FFP (2.11%). TTI positivity remained the leading cause, responsible for 85.2% of discarded WB units.^21^

Compared with national and international centers, our wastage pattern was distinctive, showing a higher proportion of discarded FFP. Unlike other hospitals where TTI-related wastage remains significant, strict pre-donation screening effectively eliminated this category. The high proportion of discarded FFP in our study may reflect an imbalance between FFP availability and utilization. As expiry was the predominant cause of wastage, lower utilization of FFP relative to its availability and challenges in accurately forecasting component requirements may have contributed to the higher wastage of FFP observed in our setting. In contrast, the minimal wastage of platelets may be attributable to their short shelf life, which necessitates close inventory monitoring, production based on anticipated clinical requirements, and maintenance of optimal inventory levels. Improved forecasting of component requirements, enhanced coordination between the blood bank and clinical services, implementation of patient blood management strategies, and timely redistribution of surplus components may help reduce expiry-related wastage.

### Strength of the study:

A major strength of this study is that it provides locally generated evidence on component utilization and wastage patterns in a tertiary care hospital, an area for which published data from Pakistan remain limited. The findings contribute to hemovigilance efforts and offer insights that may inform transfusion practices in resource-limited healthcare settings. In addition to reducing the availability of a lifesaving resource, blood component wastage imposes a substantial economic burden through collection, processing, testing, storage, and disposal costs. Enhanced coordination between the blood bank and clinical services, regular audits, and implementation of patient blood management strategies may help improve efficiency and reduce expiry-related blood component wastage services.

### Limitations:

This study was conducted at a single tertiary care hospital, which may limit the generalizability of findings to other settings. Data were collected retrospectively from blood bank records, restricting analysis of the clinical appropriateness of transfusion requests. Furthermore, detailed patient clinical information was not available, and factors associated with blood utilization and wastage could not be explored through analytical statistical methods.

## CONCLUSION

Whole blood and RCCs were the most commonly utilized components, mainly in intensive care, obstetric, and medical wards, indicating limited adoption of component therapy. The overall wastage rate exceeded international benchmarks, with FFP contributing the largest share, primarily due to expiry of units. Regular audits, rational ordering, and strict adherence to transfusion guidelines are essential to optimize utilisation. Implementation of a Maximum Surgical Blood Ordering Schedule (MSBOS), improved inventory management, and coordination with nearby blood banks are recommended to minimize wastage and ensure timely availability of this life-saving resource during emergencies.

### Acknowledgment:

The authors would like to acknowledge Prof. Sania Shuja, Head of the Department of Pathology, for her valuable guidance and writing assistance in revising the research proposal for IRB submission. Her unwavering support, encouragement, and facilitation as the departmental head greatly contributed to the successful execution of this research work.

### Authors` Contribution:

**AA**: Principal Investigator, Conceived and designed the study; prepared the synopsis; collected and analyzed data; drafted and revised the manuscript. She is also responsible for the integrity of the research and the accuracy of the data.

**NS:** Critically reviewed and approved the final version of the manuscript for publication.

**ZM AM:** Literature search, Contributed to data collection. Critical Review.

All authors have read and approved the final version of the manuscript.

***Grant Support & Financial Disclosures:*** None.
